# The Biological Effects of IL-21 Signaling on B-Cell-Mediated Responses in Organ Transplantation

**DOI:** 10.3389/fimmu.2016.00319

**Published:** 2016-08-23

**Authors:** Yongkang Wu, Nicole M. van Besouw, Yunying Shi, Martin J. Hoogduijn, Lanlan Wang, Carla C. Baan

**Affiliations:** ^1^Department of Laboratory Medicine, West China Hospital, Sichuan University, Chengdu, China; ^2^Sector Nephrology & Transplantation, Department of Internal Medicine, Erasmus MC, University Medical Center Rotterdam, Rotterdam, Netherlands; ^3^Department of Nephrology, West China Hospital, Sichuan University, Chengdu, China

**Keywords:** IL-21, IL-21 receptor, JAK/STAT, B-cell, organ transplantation, rejection

## Abstract

Antibody-mediated rejection has emerged as one of the major issues limiting the success of organ transplantation. It exerts a highly negative impact on graft function and outcome, and effective treatment is lacking. The triggers for antibody development, and the mechanisms leading to graft dysfunction and failure, are incompletely understood. The production of antibodies is dependent on instructions from various immunocytes including CD4 T-helper cells that secrete interleukin (IL)-21 and interact with antigen-specific B-cells *via* costimulatory molecules. In this article, we discuss the role of IL-21 in the activation and differentiation of B-cells and consider the mechanisms of IL-21 and B-cell interaction. An improved understanding of the biological mechanisms involved in antibody-mediated complications after organ transplantation could lead to the development of novel therapeutic strategies, which control humoral alloreactivity, potentially preventing and treating graft-threatening antibody-mediated rejection.

## Introduction

Antibody-mediated rejection remains an important barrier to improving long-term survival after solid organ transplantation (1–3). In cellular rejection, graft injury is due to direct cytotoxic activity of immune cells against graft parenchymal tissue. Antibody-mediated rejection, in contrast, is characterized by graft damage induced by circulating alloantibodies. Alloantibodies are produced by activated B-cells in response to antigen, costimulation, and cytokines such as interleukin (IL)-21 (4, 5).

Interleukin-21 was discovered by Parrish-Novak et al. using a functional cloning approach based on expression of the IL-21 receptor (IL-21R) gene and is located at chromosome 4 on position q26–q27 (6). The common γ-chain (γc) is a component of the IL-21R complex. IL-21 binding to the IL-21R/γc results in signaling *via* the JAK/STAT pathway (6, 7). This cytokine, a four-α-helix bundle, is a typical family I cytokine with broad pleiotropic actions and is primarily produced by T follicular helper cells (Tfh), Th17, and natural killer T-cells, rather than being generally produced by most tissue cells (6, 8, 9). IL-21 controls the activation, proliferation, differentiation, cytotoxicity, and survival of various target immune cells (10, 11). It is also important for the generation of B-cell responses in germinal centers resulting in isotype switching, affinity maturation, antibody production, and development of B-cells (12, 13). In particular, IL-21-mediated actions by Tfh cells are required for efficient antibody responses. The effectors and immune regulatory functions of IL-21 are mediated by binding to target B-cell surface receptors, which consist of α-chain and the γc that is shared with IL-2, IL-4, IL-7, IL-9, and IL-15 receptors (10, 14, 15).

Antibody-mediated (“humoral”) rejection is a key cause of graft dysfunction and failure after organ transplantation (1, 16, 17) with 30–50% of failed allografts affected (18–20). Immunohistochemical and gene expression studies have shown that a large number of B-cells infiltrate the rejected allograft (18, 21–24), contributing to anti-donor responses.

Identifying the role of IL-21-mediated B-cell activation and differentiation pathways is critical for understanding the signaling pathways that underlie antibody-mediated rejection. In this review, we discuss the potential role of IL-21 on B-cells after organ transplantation.

## IL-21 Signaling Pathway in B-Cells

The IL-21R is expressed by human naive B-cells, memory B-cells, germinal center B-cells (14), and as shown recently, plasma cells (25). IL-21R is upregulated on human memory B-cells after activation by anti-CD40 mAb (14).

**Figure 1:**
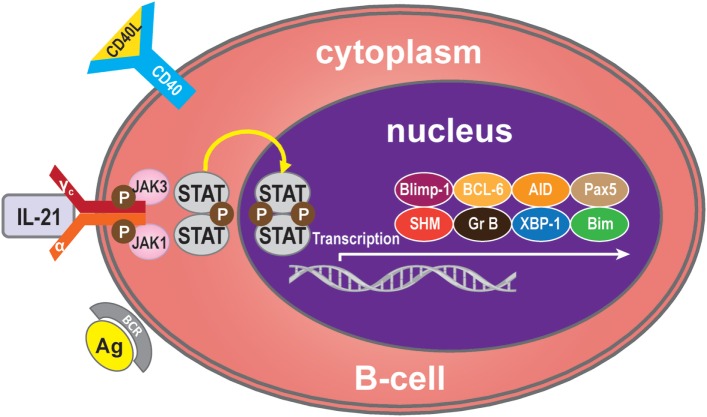
**IL-21 signaling pathway**. Many molecules participate in the IL-21 signaling pathway in B-cells, but the main molecules are IL-21R, JAK, and STAT to activate transcription of Blimp-1, BCL-6, AID, Pax5, SHM, granzyme B, XBP-1, and Bim. Generally, IL-21 binds with the IL-21R of B-cells to trigger signaling pathways. The JAK and STAT family molecules are activated in turn, while the balance of the transcription factors Blimp-1 and BCL-6 control the maturation B-cell.

Binding of IL-21 with IL-21R/γc triggers the catalytic activation of JAK1 and JAK3. This causes phosphorylation of tyrosine residues on IL-21R/γc, providing docking sites for STAT proteins and other signaling molecules (26). On recruitment, STATs are phosphorylated and form homodimers or heterodimers, which translocate into the nucleus and modulate expression of the target genes (27), which regulate B-cells, such as B-cell-induced maturation protein-1 (Blimp-1) (28), B-cell lymphoma (BCL)-6 (29), activation-induced cytidine deaminase (AID) (30), granzyme (31), somatic hypermutation (SHM) (32), paired box 5 (Pax5) (33), X-box-binding protein 1 (XBP-1) (34), and Bim (35). IL-21 mediates B-cell proliferation, immunoglobulin (Ig) production, and apoptotic functions mainly through the potent effects of STAT3 and/or STAT1 activation but also, to a lesser extent, through STAT4 and STAT5 (36–39) (Figure 1).

## B-Cell Activation and Differentiation

B-cell receptor (BCR) ligation triggers activation of multiple downstream molecules. Burton’s tyrosine kinase (Btk), one of the downstream products of the BCR signaling pathway, selectively regulates IL-21-induced STAT1 phosphorylation and translocation in the nucleus. Btk deficiency is associated with arrested cell development at the pre-B-cell stage. In addition, Btk is involved in cytokine-controlled B cell activation. In concert with IL-21, CD40, and B-cell activating factor (BAFF), this kinase mediates the crosstalk with cytokine pathways through regulation of IL-21-induced phosphorylation of STAT1 (25). IL-21 and CD40L collaborate to synergistically promote Blimp-1 activation and plasma cell differentiation (28). CD40L alone has no direct effect on Blimp-1, but it greatly augments the IL-21-triggered JAK-STAT signaling. During this phase, STAT3 plays a more significant role than STAT1, because STAT3 mutations dramatically reduce the number of memory B-cells and abolish the ability of differentiation of naive B-cells into plasma cells (10). In contrast, STAT1 deficiency has no effect on memory B-cell formation *in vivo*. Thus, STAT3 is essential for the generation of effector memory B-cells from naive precursors (40). In addition, treatment with CD40L enhances the ability of STAT3 to upregulate Blimp-1 by removing BCL-6, which is a potent inhibitor of Blimp-1 expression. It has been speculated that IL-21 induces Blimp-1 and BCL-6 to regulate isotype-switched B-cells (41). Blimp-1 is a transcription factor and involved in plasma cell formation and maturation (42). Importantly, IL-21 costimulation upregulates expression of Blimp-1 (43). Consistent with this, IL-21-driven plasma cell differentiation from both naive blood B-cells and from memory B-cells are preceded by induction of Blimp-1 upregulation. Blimp-1 initiates plasma cell differentiation by downregulating MHC, CIITA, Pax5, and c-myc expression (33, 44, 45) and by inducing XBP-1 expression (46, 47). Blimp-1 level is very low when BCL-6 is over-expressed in B-cells (48). BCL-6 may block plasma cell differentiation due to downregulation of Blimp-1 (49). BCL-6 also can control B-cell development by BTB and RD2, two molecules that repress distinct functional effects of B-cells during the germinal centers reaction. BTB is required for B-cell survival and proliferation, while RD2 might be important for the prevention of terminal B-cell differentiation (50).

**Figure 2:**
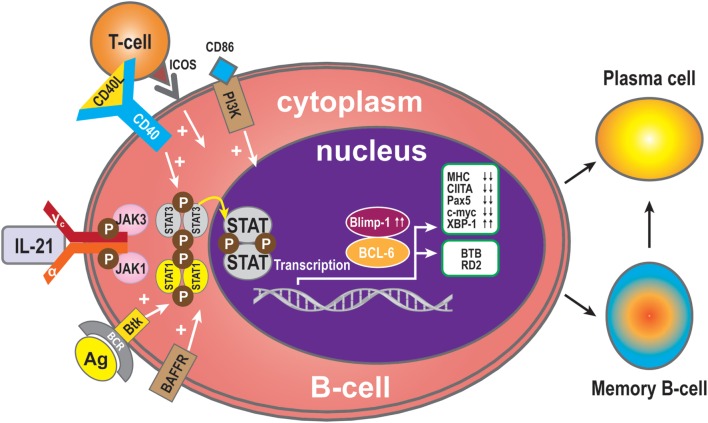
**B-cell activation and differentiation**. Plasma cells are the main executors of B-cell regulation by the IL-21signal pathway. STAT3 is the dominant member of the STAT family in this respect. Transcription Blimp-1 has a positive role and BCL-6 a negative role in plasma cell maturation. Additionally, CD40L, ICOS, CD86, and BAFF can promote B-cell differentiation to plasma cells, while MHC, CIITA, Pax5, and c-myc are switched off during B-cell differentiation to plasma cells or memory B-cells, and XBP-1 is induced. The transcription factor BCL-6 activates BTB, which is required for cell survival and proliferation, while RD2 prevents terminal differentiation of B-cells.

Since IL-21 activates STAT3 in B-cells, this may indicate that activation of STAT3 in human B-cells is pivotal for the induction of Blimp-1 expression and plasma cell differentiation (11, 40). It has been reported that IL-21-dependent CD86 upregulation is reliant on STAT3 phosphorylation and PI3K, revealing unexpected roles for these pathways in IL-21-mediated B-cell responses (51). In addition, IL-21 drives humoral immune responses *via* STAT3-dependent induction of the transcription factors required for plasma cell generation (52). These authors reported that IL-21*via* STAT3 sensitizes B-cells to the stimulatory effects of IL-2. Thus, IL-2 plays an adjunctive role in IL-21-induced B-cell differentiation. An absence of this secondary effect of IL-21 may amplify humoral immunodeficiency in patients with mutations in STAT3 and IL-21R due to impaired responsiveness to IL-21. In concert, IL-21 and BAFF stimulate and may maintain humoral immunity in humans (53). BAFF has the ability to substitute for CD40L activity with regards to IL-21 costimulation and differentiation of memory B-cells present in spleen (53) (Figure 2).

## Immunoglobulin Production

**Figure 3:**
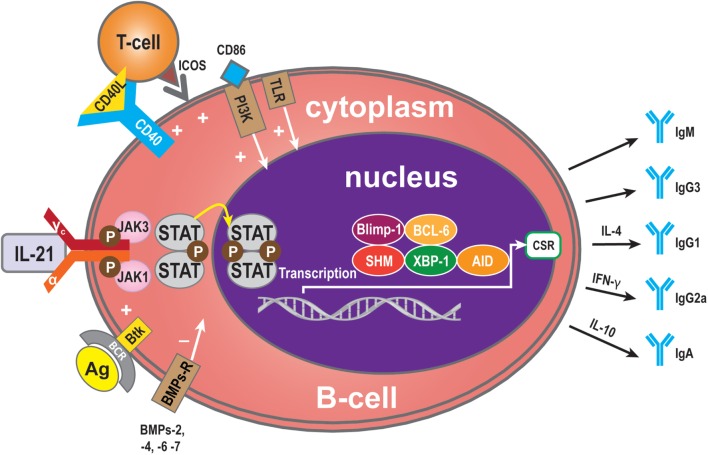
**Immunoglobulin (Ig) production**. Ig is produced by plasma cells, so the signaling pathway for mediation of Ig production is similar to that for IL-21-mediated plasma cell maturation. Some molecules, however, have a specific role in Ig production: BMP-2, -4, -6, and -7 may exert a negative influence and Btk a positive influence. In addition, some cytokines contribute to Ig CSR. IL-4 can induce to IgG1 formation, IFN-γ to IgG2a, and IL-10 to IgA.

Critical sites for the generation of antibody responses are the germinal centers in lymphoid follicles present in lymph nodes that also have been identified in transplanted organs (4, 54) where antigen-primed B-cells interact with T-cells, most of which are Tfh cells secreting IL-21. The B-cells are driven to undergo Ig isotype switching, with SHM and secretion of high affinity antibodies (12, 55–58). Bryant et al. reported that IL-21 stimulates naive B-cells to mainly produce IgM^+^ B-cells, while low frequencies of IgG and IgA secreted B-cells were also detected (59). When IgG was produced, IL-21 favors naive B-cells to develop into IgG1- and IgG3-secreting B-cells (56, 57, 59–62). It has been reported that IgM-specific Abs targeting BCR and IL-21 costimulation also induce the expression of AID (63, 64). Interestingly, although AID catalyzes both class switch recombination (CSR) and SHM, only CSR is induced in naive human B-cells after stimulation by IL-21 and anti-CD40 (45, 47, 60, 65). The C-terminal of AID is required for CSR but not for SHM (65, 66), and it has been postulated that IL-21 induces AID activity only at the C terminus. Multiple studies have shown that IL-21 causes CSR of CD40-stimulated human naive splenic IgM^+^ B-cells to IgG1 and IgG3, and CSR of CD40-stimulated cord blood B-cells to IgA (47, 60). As well as the molecules described above, among the group of cytokines called bone morphogenetic proteins (BMPs) (67), BMP-2, -4, -6, and -7 inhibit CD40L/IL-21-induced production of IgM, IgG, and IgA. In memory B-cells, BMP-6 upregulated expression of DNA-binding protein inhibitor genes, but potently inhibited CD40L/IL-21-induced upregulation of the transcription factor XBP-1 (34). This factor is crucial for final stage in plasma cell differentiation (34). As described above, Btk is an efficient propagator of IL-21 signaling, critical for CSR in human B-cells and secretion of Ig (25). Additionally, the outcome of IL-21-mediated Ig secretion depends on the presence of IL-4 and IL-10, which influence the outcome of IL-21-mediated CSR. IL-10 acts synergistically with IL-21 to induce secretion of IgA by CD40L-stimulated human B-cells, whereas IL-4 has an inhibitory effect (47). As shown by the group of Bromberg, IL-10 deficiency in B-cells prevents transplantation tolerance, resulting in decreased follicular immune regulatory CD4^+^ T-cells, a recently identified T cell subset, and increased IL-21 expression by Tfh cells in the B-cell and T-cell marginal zones (68). This has implications for our understanding of the mechanisms involved in tolerance and show at the same time that B cells play pivotal roles in the induction of this immune phenomenon (68). Interestingly, as with IL-21, IL-10, in combination with toll-like receptor (TLR), signaling also enhances phosphorylation of STAT3, resulting in increased IgG production. Hence, IL-21 and IL-10 increase the activity of the TLR–MyD88–STAT3 pathway in human B-cells by enhancing Ig production stimulated by STAT3 phosphorylation (69) (Figure 3).

## Regulatory B (B10) Cells

**Figure 4:**
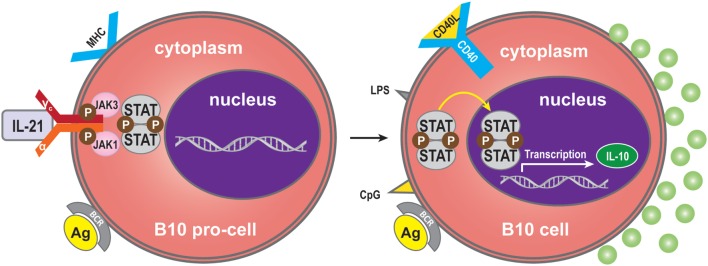
**Regulatory B (B10) cells**. IL-21 binding to IL-21R expressed on B10 pro-cells may trigger B10 pro-cell maturation to B10 cells. Synergistically with MHC-II, LPS, CpG, or CD40 ligation stimulation can induce this cell subset to produce IL-10.

Interleukin-21 may also modulate the immune response by immune-dampening regulatory mechanisms. One of these is performed by B10 cells, named for their ability to produce abundant IL-10 (70). Expression of IL-10 is a common characteristic of regulatory immune cells, and B10 cells are thus referred to as regulatory B-cells (71, 72). The B10 cell subset represents <1% of peripheral blood B-cells in humans (73). A high proportion of peripheral B10 cells and progenitor (pro)-B10 are present in the CD24^hi^CD27^+^ B-cell subset, and approximately 60% also express CD38 (73). B10 progenitors and B10 cells have been identified in human (73). *Ex vivo*, human B10 progenitors can be driven to develop into B10 cells by lipopolysaccharide (LPS) or 5′-C-phosphate-G-3′ (CpG), or by CD40 ligation. *In vitro*, IL-21/CD40-receptor signaling pathways can promote the development and expansion of B10 cells by four million-fold to suppress the immune response. IL-21R signaling, together with major histocompatibility complex class II and CD40 cognate, interacts with CD4^+^ T-cells and although not required for B10 cell development, are necessary for B10 cell effector functions that result in antigen-specific responses. Interestingly, BCR ligation augments human B-cell IL-10 responses to CpG (74). Whether human B10 cells develop into antibody-secreting cells, or enter the memory B10 cell subset, remains to be determined (75). B10 cells may represent a subset, which is similar to regulatory T-cells (76) (Figure 4).

B10 cells are able to control the immune response, but an excessive reaction from these cells may also promote tumor cell growth or chronic infection (77). It is possible that regulatory fine tuning by B-cells and IL-21 production by T-cells might be a key factor in maintaining immune tolerance (78). Most investigations of B10 cells have concentrated on autoimmune diseases (79), but a few have assessed their role in transplantation (80). A mouse islet T-cell transplantation study has demonstrated that B10 cells control immune responses (81).

## B-Cell Apoptosis Mediated by IL-21

**Figure 5:**
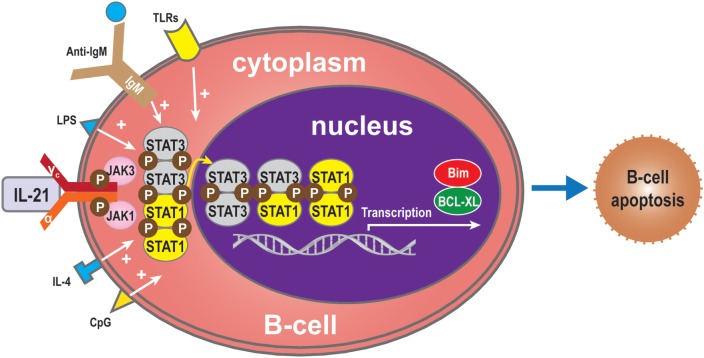
**B-cell apoptosis**. IL-21 can also induce apoptosis of B-cells when activated by LPS, TLRs, CpG, anti-IgM, or IL-4 in the absence of T-cell signals. In the absence of such molecules, the balance between STAT1 and STAT3 regulates B-cell apoptosis *via* the IL-21 signaling pathway. STAT1 induces cell death, while, conversely, STAT3 exerts an anti-apoptotic effect. Bim also plays an apoptotic role and BCL-XL an anti-apoptotic role.

The effects of IL-21 on B cells depend on the costimulatory signals that are received. In the absence of signal from a T cell (such as the T cell engaging CD40), BCR activation is required for IL-21-mediated B cell apoptosis (15, 29, 35). The balance between STAT1 and STAT3 is critical for IL-21-induced B-cell apoptosis in the IL-21 signaling pathway. STAT1 mainly acts in cell cycle arrest and apoptotic cell death (45, 47, 82, 83). By contrast, STAT3 mostly exerts an anti-apoptotic effect, especially in numerous malignancies where it is constitutively active (83). In some circumstances, IL-21 can induce apoptosis of B-cells activated *via* signals through the TLR, LPS, CpG, anti-IgM, and IL-4 (11, 15). Complete protection from IL-21-mediated apoptosis was not inhibited by other molecules involved in apoptotic pathways. Functional studies have demonstrated that IL-21 substantially inhibited proliferation and Bim-dependent apoptosis of activated mouse B-cells (47). Hagn et al. reported that CpG together with IL-21 may enhance their apoptosis-inducing and immunogenizing effects (84). It is therefore possible that combining CpG with IL-21 could more effectively induce apoptosis in B-cells than CpG or IL-21 alone. Furthermore, IL-21 can inhibit B-cell proliferation when receiving a strong signal *via* TLR while preventing apoptosis of B-cells *via* upregulation of B-cell leukemia/lymphoma-X linked (BCL-XL), an anti-apoptotic protein of the BCL-2 homology 3 (BH3) family (11, 85) (Figure 5). From this viewpoint, IL-21 appears to act as an immunosuppressive cytokine on B-cells. This finding indicates that the apoptotic effects of IL-21 may only be relevant in situations where a humoral immune response is improperly triggered, thereby shutting down at least one arm of the immune system before extensive damage is done (7).

## Granzyme B Production by B-Cells

**Figure 6:**
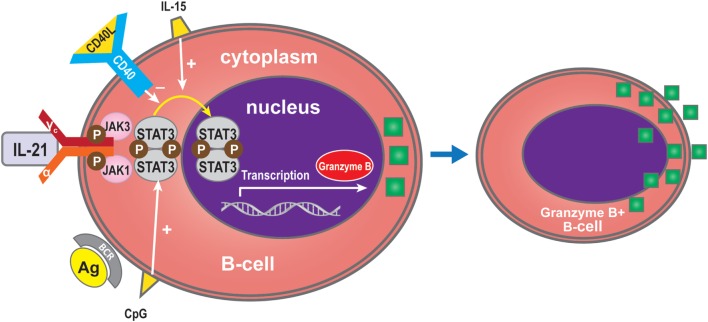
**Granzyme B expression by B-cells**. IL-21 can induce BCR-stimulated B-cells to differentiate into granzyme B (GrB), an effect which is dependent on STAT3 and which is promoted by IL-15 or CpG. CD40 inhibits differentiation into GrB-cells.

Interleukin-21 can induce BCR-stimulated human B-cells to differentiate into granzyme B-expressing cytotoxic cells (GrB) in a STAT3-dependent manner in the absence of a CD40 signal (31, 77, 86–88). GrB^+^ B-cell numbers are dependent on IL-21 production, and increasing doses of anti-IL-21 decreased the number of GrB-expressing B-cells in co-culture systems (78). The increase in GrB^+^ B-cells in the circulation of tolerant recipients may be due to a direct effect of IL-21 (78). GrB secreted by B cells may play a key role in the regulation of immune responses (78, 89). Xu et al. showed that IL-21 initially triggers transcription of the GrB gene in B-cells, while STAT3 is required for GrB synthesis in PCs activated by IL-21 and IL-15. The defect in GrB formation in STAT3-deficient B-cells might arise from a lack of cell proliferation and differentiation (88). Recent *in vitro* studies have indicated that CD40 signaling in B-cells inhibits their differentiation into GrB^+^ cells (31, 77) (Figure 6).

## IL-21 as a Possible Player in Alloreactivity after Transplantation

Antibody-mediated rejection is a major problem after organ transplantation mediated by anti-HLA antibodies and donor-specific antibodies (DSA). This poorly defined alloimmune response is refractory to treatment with conventional immunosuppression (1). From our recent studies, we know that in this process, B-cells can be activated by IL-21-producing Tfh cells and differentiate into Ig-producing plasma cells. We reported that these Tfh cells as well as B-cells infiltrate the allograft during rejection and colocalize in follicular-like structures in the transplanted kidney (4, 18). These tide clusters of T and B-cells form highly organized lymphoid structures named tertiary lymphoid organs (TLOs). Associations between the presence of these TLO and poor graft outcome have been reported (90–92). In contrast, Xu et al. reported that IL-17, and not IL-21, is responsible for lymphoid neogenesis. Therefore, they suggested that Th17, but not Tfh, cells could play a role in the process of lymphoid neogenesis (93). It is likely that infiltrated and organized T and B-cells contribute to the anti-donor response by antigen presentation of B cells and by help of Tfh cells to the infiltrated B-cells. Besides IL-21, the capacity of Tfh cells to provide help to B-cells depends upon the acquisition of molecules that are known to play functional roles in T-cell–B-cell interactions, such as CD40 ligand, inducible co-stimulator (ICOS), and programed death 1 (PD-1) (18, 33, 94, 95).

In organ transplantation, specifically targeting B-cells to decrease plasma cell differentiation by either IL-21-dependent B cell apoptosis or IL-21R blockade may provide novel approaches for the prevention of the development of *de novo* DSA and treatment of antibody-mediated rejection.

The first approach is speculative and based on the finding that IL-21 induces B-cell apoptosis when costimulation signals are absent (15, 29, 35). At the same time, IL-21 might stimulate the cytolytic functions of alloantigen activated CD8 T cells, the aggressors in acute rejection (96, 97). Therefore, we should be careful with IL-21 cytokine treatment. This strategy should first be tested in experimental animal models by using various concentrations of IL-21 to define if B cell apoptosis and T cell cytotoxicity rely on the same or different concentrations of IL-21. This knowledge is helpful to better understand the role of IL-21 in B-cell-mediated immune processes such as apoptotic cell death.

The second approach could be blockade of the IL-21 pathway proven to affect the production of pathogenic immunoglobulins in animal models of autoimmune diseases. In these studies, blockade of the IL-21R signaling pathway reduced B-cell-mediated diseases (98). Also, in a mouse model of islet transplantation, mIL-21R-Fc combined with CTLA-4-Ig diminished T-cell and B-cell effector functions, and tolerance was induced in a subgroup of treated animals (99). It is critical to determine whether neutralizing the IL-21 function also inhibits production of anti-HLA antibodies and DSA in organ transplant recipients. So far, such studies have not been conducted, but based on the biological functions of IL-21, the promising findings in animal models for autoimmune diseases and *in vitro* studies, targeting the IL-21 pathway could be expected to reduce the incidence of antibody-mediated alloreactivity. Our studies using peripheral T-cells and B-cells derived from kidney transplant patients showed that the interaction between IL-21-producing Tfh cells and B-cells could be inhibited by an IL-21 receptor antagonist. In these co-cultures, B-cell differentiation and IgM and IgG production were diminished (4). We believe that IL-21-producing Tfh cells play a dominant role in alloreactivity and should be targeted by novel immunosuppressive agents.

**Figure 7:**
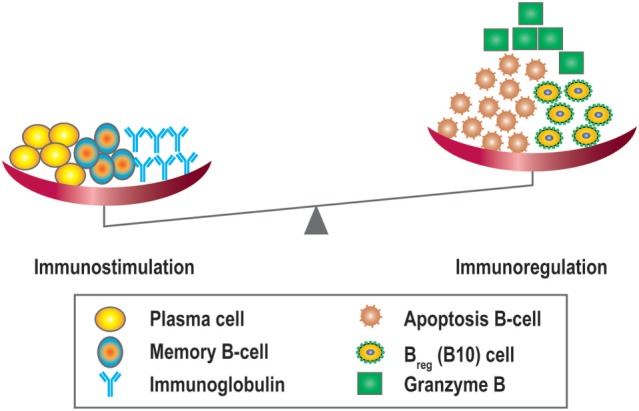
**Overview of the immunostimulatory and immunoregulatory effects of IL-21**. IL-21 promotes B-cell proliferation, plasma cell differentiation, B-cell memory, and Ig class switching, and is also important for the development of IL-10 + regulatory B-cells (Bregs/B10), B-cell apoptosis, and granzyme B producing cells.

Like many other cytokines, IL-21 has multiple functions. In addition to its actions in B-cell apoptosis and differentiation it also drives regulatory B10 responses. These cells have been shown to suppress T-cell-mediated rejection induced by mismatched MHC molecules and prolong allogeneic islet T-cell survival, suggesting a potential regulatory role for B10 cells in organ transplantation (80, 81). Since IL-21 can promote regulatory B10 cell proliferation, harnessing the anti-inflammatory properties of B10 cells by anti-IL-21 agents could potentially stimulate antibody-mediated rejection and promote a less favorable tolerogeneic environment by modulating the plasma cell/Breg (B10) balance (68) (Figure 7). Recently, another type of Bregs was described, which could be inhibited by anti-IL-21 treatment. The number of GrB-producing B-cells with regulatory properties was significantly higher in tolerant patients compared to patients with stable graft function (78). This observation suggests that targeting the IL-21R pathway with immunosuppressive agents may harness this cell population. Data in this area, however, remain sparse.

## Conclusion

In general, IL-21 promotes humoral immunity, and IL-21 blockade may attenuate B-cell hyperactivity in which also costimulatory signals are involved. However, B-cells may have a dual effect, acting both as a driver and as a regulator of the immune system (78, 79, 100). In B-cells that recognize Ag and receive T-cell help, IL-21 induces survival, proliferation, isotype switching, and differentiation to Ig-secreting plasma cells or GrB-producing B-cells. B-cells can also cause cell death or, in the form of regulatory B10 cells, can induce autoimmunity if they receive a strong signal *via* BCR, or *via* TLR, and IL-21 costimulation. An equilibrium between effector and suppresser cells is necessary to maintain B-cell homeostasis and the immune balance, especially for the prevention of antibody-mediated transplantation rejection. Future studies should focus on elucidating details of the signaling cascades and downstream changes in gene and protein expression within B-cells in response to IL-21, either alone or in combination with other molecules. This knowledge may ultimately lead to an effective therapeutic strategy to overcome antibody-mediated rejection following transplantation, particularly by targeting the differentiation of B-cells into plasma cells *via* IL-21 signaling pathways.

## Author Contributions

YW, NB, YS, MH, LW, and CB researched the literature and wrote the review.

## Conflict of Interest Statement

The authors declare that the research was conducted in the absence of any commercial or financial relationships that could be construed as a potential conflict of interest.

## Abbreviations

AID, activation-induced cytidine deaminase; BAFF, B-cell activating factor; BCL-6, B-cell lymphoma-6; Blimp-1, B lymphocyte-induced maturation protein-1; BMPs, bone morphogenetic proteins; Btk, Bruton’s tyrosine kinase; BTB, also named POZ, pox virus and zinc finger; CpG, 5′-C-phosphate-G-3′; CSR, class switch recombination; GrB, granzyme B-cell; Ig, immunoglobulin; IL-21, interleukin-21; IL-21R, IL-21 receptor; LPS, lipopolysaccharide; mAb, monoclonal antibody; RD2, the second repression domain; Tfh, follicular T helper cells; TLO, tertiary lymphoid organ; TLR, toll-like receptor; XBP-1, X-box-binding protein-1; γc, common γ chain.
